# Does Exposure to Foreign Culture Influence Creativity? Maybe It's Not Only Due to Concept Expansion

**DOI:** 10.3389/fpsyg.2019.00537

**Published:** 2019-04-11

**Authors:** Liu Tan, Xiaoqin Wang, Chanyu Guo, Rongcan Zeng, Ting Zhou, Guikang Cao

**Affiliations:** ^1^Key Laboratory of Cognition and Personality, Ministry of Education, Southwest University, Chongqing, China; ^2^Faculty of Psychology, Southwest University, Chongqing, China; ^3^Mental Health Education Center, Henan Normal University, Xinxiang, China

**Keywords:** multicultural experience, creativity, physiological awaken, culture shock, cultural priming paradigm

## Abstract

Multicultural experience refers to those experiences gained through individuals' contact with other cultures. This study focused on exploring whether knowledge of different cultures can improve creative performance-and also how multicultural experiences influenced this performance through changes in individual's physiological mechanisms. Study 1 explored the influence of different cultural priming on creative story-writing tasks. Eighty-nine Chinese college students were randomly assigned to 4 conditions: sole American culture, dual cultures, sole Chinese culture or control condition, and made to watch 45 min slides with cultural elements—including pictures, music and videos,—and then they were asked to complete the creative story-writing task. The results showed that American culture priming group's score was significantly higher than the control condition with regards to the uniqueness and novelty of the creative story-writing task. Study 2 was aimed at exploring the relationship between physiological arousal levels induced by different cultural and creative performance. We divided the whole experiment into five stages,—including the baseline, picture, listening to music, watching video, and completing creative tasks. Through Biofeedback measurement, we recorded the physiological indexes of participants in different groups in every stage, including skin conductance, thermal, electroencephalographic, and heart rate. The results showed that contacting with foreign cultures would increase individuals' physiological arousal level and brain activity, which contributed to the following creative task.

## Introduction

Creativity, which refers to the generation of useful and appropriate new ideas (Dong et al., 2016), is one of the most important capabilities of the twenty-first century (Fink et al., 2014; Miron-Spektor and Beenen, 2015). There are two common elements in the definition of creativity, namely novelty and applicability (Sternberg et al., 1999; Runco and Jaeger, 2012). The measurements of creativity include the cognitive process of the creativity and creative support, such as the measurement of creative performance through problem solving tasks, remote association task, story-writing task and divergent thinking tasks (Leung, 2008) Creativity depends on many factors including environment, personality, cognition and motivation and so on (Amabile, 1996; Csikszentmihalyi, 1996; Sawyer, 2006; De Dreu, 2010; Chua et al., 2012, 2014). Zha et al. (2006) also proposed that culture could contribute to individual creativity in respect of the definition and evaluation of creativity. However, whether different cultures have different influence on creativity and the psychological mechanism by which culture influences creativity all remain to be explored.

Recent researches have shown that multicultural experience could enhance creativity (Presbitero, 2016; Falavarjani and Irandust, 2017). Multicultural experience refers to all direct and indirect experiences gained by individuals when they communicate or connect with members or elements of other cultures (Leung, 2008; Aytug et al., 2018). Due to the diversity of cultures, multicultural experience should include not only the experience brought by exposure to cultures of different countries, but also the experience brought by exposure to cultures of different nations and regions. Chang et al. (2017) suggested that multicultural experiences not only provide individuals with opportunities to learn new concepts and knowledge, but often require the establishment of innovative frameworks, which help to solve the incongruity when the idea of new learning is incompatible with individuals' prior knowledge structures (Chang et al., 2017). Studies also have shown that exposure to working groups that include members of multiple cultures or contacting a diverse range of perspectives generated by groups, are positively correlated with the development of creative potential (Guimerà et al., 2005; Kurpis and Hunter, 2016; Sparkman et al., 2016). It may be that this exposure increases tolerance for the mixed views of organizations and teams. Other research shows that individuals who speak two kinds of language have higher levels of creative performance than those who speak one language (Lambert et al., 1973). In addition, previous evidence showed that race diverse groups, such as first or second generation immigrants, were more creative (Simonton, 1999), which was evaluated by multidimensional indexes, such as verbal fluency, flexibility and originality (Chang et al., 2014). Chang et al. (2015) found that the children of parents from two different cultures would have better performance in general fields and special fields mathematics creativity. Tendayi Viki and Williams (2014) found that the bicultural experience in the family is conducive to the creativity of children who belong to mixed race group. In addition, more and more research shows that studying abroad could stimulate individuals' creativity (Hu et al., 2017). Besides, the results found that people who lived abroad could solve the Duncker Candle Problem much more easily than those who did not, while the time spent living abroad significantly predicted creative solutions. However, the time spent on traveling abroad before had no correlation with individual creativity (Galinsky et al., 2006; Falavarjani and Irandust, 2017). Shi et al. (2012) found that the disintegration of multicultural experience general knowledge structure improved the access to knowledge which was difficult to obtain in conventional cases. Yang and Li (2015) suggested that individuals with multicultural experience were more likely to obtain complex construct cultural cognition and improved their cognitive complexity. And research also showed that multicultural experience would enable individuals to adopt the global processing cognitive styles in information processing (Yang and Wan, 2012; Yang, 2014). All of these studies showed that creativity was promoted to a certain extent when an individual was in one or more new cultures, and previous studies have indicated that this was most likely due to the fact that when an individual experienced a multicultural environment, the cognitive process would be changed, which might enhance individuals' creativity.

For individuals without experience abroad, studies also found that the multicultural experience could make the participants tend to the whole processing of information, and properly has the significant positive correlation with creative problem solving (Gaither C. J. et al., 2015). It can also have a positive impact on verbal creative tasks. Furtherly, the scores of novelty of the AUT task and story-writing task of the participants in the American culture priming group were significantly higher than those in the Chinese culture group and the control group, and multiracial participants who were primed with flexible multiracial identities performed significantly better on RAT tasks than non-primed multiracial participants and primed mono-ethnic participants. These studies have shown that even if individuals do not live in a new cultural environment, they could still get multicultural experience by culture priming, and the promotion effect on creativity might still exist, which proved the effectiveness of the cultural initiation paradigm to some extent.

Leung and Chiu (2010) using cultural priming paradigm, found the multicultural experience could help to improve individuals' creativity. According to researchers exposed to different cultures, the individuals' concepts would be expanded, so as to improve the creativity of the individuals. However, Leung only found this phenomenon under dual cultural conditions, and this point of view is obviously not enough to explain all the experimental results. In Leung's study, participants only exposed to Chinese cultural elements were actually stimulated by new cultural elements and gained multicultural experience. However, their creativity was not improved, so the influence of multicultural experience on creativity was not necessarily due to concept expansion. In addition, on account of the difference in mainstream culture and the disparity in national development level, American college students' degree of interest and familiarity with Chinese culture is greatly different from that of Chinese college students' degree of interest and familiarity with American culture, which leads to different results in the influence on creativity. Therefore, we speculate that since this experiment's participants were all college students in the United States, different results would be obtained if the college students in China participated in the experiment. We speculate that since the participants used in this experiment are American college students, if the subjects were replaced by participants from another cultural environment, the results might be different. Because of the difference in mainstream culture, as well as the difference in the two countries level of development, American students' interest and familiarity in Chinese culture may be different than Chinese students', leading to the change on the creative ability also could produce different results. Therefore, we would test this assumption in study 1.

Studies have shown that when individuals are separated from the environment or customs they used to live in, they would feel a sense of physical and emotional maladjustment which was brought by cultural shock caused by the new environment (Junaid and Pertiwi, 2017). Cultural shock refers to the psychological reaction when most people are exposed to a new culture that they have never experienced before. Different cultural shocks can lead to different psychological responses, such as anxiety, surprise and confusion when people enter the new environment (Kristian, 2013). Cultural shock may have some positive emotions, such as surprise, excitement, careful and good social interaction and adaptation of life changes. These experiences are likely to improve individual physiological activation level.

Clark et al. (1993) pointed out that high creativity individuals has faster synapses activities and more abundant chemical composition of neurons, so that there may be a more complex neural model underlying the information processing of high creativity individuals. He also pointed out that high creativity alpha waves of electrical activity in the brain maintained more durable and input more quickly, therefore, they had better effect on learning and memory. According to Martindale et al.'s (1996) research, higher creative people have stronger intense skin potential responses to tone stimulation than lower creative people did, and the underlying skin response is covariant with cortical activation levels. Previous study about creative individuals showed that the amplitude of the pre-frontal cortex wave was higher in the verbal association and in the imagination task, while the amplitude of the frontal lobe wave was higher in the imagination task (Hudspith et al., 1985). In researches of EEG on divergent thinking and convergent thinking, Jausovec and Jausovec (2000) found that individuals with high creativity have a higher wave power. The investigation of the cortical activity in remote association task, which has three conditions: the classic remote association task, simple associative task and eye-opening rest, showed that the power value of the β2 (20–30 Hz) distant association task's worm 2 (20–30 Hz) band were significantly increased in various brain regions compared with the other two tasks. The power of θ1 (4–6 Hz) increased significantly in the frontal cortex of the brain; and the α wave (8–13 Hz) was significantly increased in the posterior region of the brain Razumnikova (2007). Carlsson et al. (2000) explored the changes of brain blood flow in alternate uses task, and found that the high creativity individuals showed significant activation in both frontal lobes of the brain. Previous studies have shown that when individuals' cognitive activities change, the physiological arousal level might be different with a series of changes. Above results suggested that creativity was closely related to brain activity and physiological activation.

Some theories suggest that creativity has a lot to do with the level of cortical activation in the brain. There are two distinct findings about the relationship between creativity and cortical activation. One opinion suggested that high creativity people have higher cortical activation levels. Individuals who were more creative got higher scores on the word association and anxiety tests than those who were less creative did. Furthermore, there was a positive correlation between basic skin conductance measurements and creativity tests in high creativity individuals (Zhou and Zhang, 2002). Another view claimed that high creativity individuals have lower levels of basic activation. Wyspianski et al. (1963) found that compared with high creativity people, the amplitude of α wave is greater in the low creativity people, which can represent the activation level of cerebral cortex. Another experimental evidence showed that the plasma levels of uric acid in the high creativity individual are low, since the plasma levels in the high creativity individual reflect the physiological activity status, which also indicates that the basic activation level of the high creativity individual is relatively low. Due to these different findings, some researchers proposed the variability of cortical activation level, and the variability of physiological activation was higher in individuals with high creativity. Studies by Martindale and Hasenfus (1978) showed that individuals with high creativity showed more variability in their skin electrocardiogram and EEG α waves. Previous studies have shown that attentional alertness level changes are accompanied by a series of physiological arousal changes (Pfaff, 2008; Smolders and de Kort, 2014). For example, the increasing of the sustained attention and the alertness level had a positive relationship with EEG low theta activity (Oken et al., 2006). Besides, there was a negative correlation between attentional alertness level and Electromyogram (EMG) and maximum fractal length (MFL) of EEG (Arjunan et al., 2009). As we could know from the research reviews above, we could conclude that multicultural experience would change individuals' cognitive process of information processing and changes in cognitive processing were usually accompanied by a series of physiological changes. Since there was a correlation between physiological arousal changes and creativity promotion, study 2 designed to explore the internal mechanism of multicultural experience to promote creativity from the perspective of physiological arousal. Therefore, in current study, we also wanted to explore the relationship between multicultural activation and physiological arousal, and the effect of activation of this physiological state on creativity.

## Study 1

To explore the influence of different cultural startup conditions (American cultural priming conditions, Chinese cultural priming conditions, American–Chinese fusion culture conditions and control conditions) on the story-writing task.

### Methods

#### Participants

The participants were selected from students of Southwest University, which were randomly assigned to such four groups. This is a between-subjects design with 20 subjects in each condition. In accordance with the Declaration of Helsinki (1991), the experiment was approved by the Academic Committee of the School of Psychology and the local ethics committee of the School of Psychology, Southwest University in China. We had obtained appropriate ethics committee approval for the research reported, and all subjects gave written informed consent before our experiment.

#### Materials

Prime conditions: American cultural priming conditions, Chinese cultural priming conditions, American–Chinese fusion culture conditions. Each condition was presented to the subjects as a slide show, which included 160 images and was played with a music background of the corresponding culture for 20 min. After that were 10 min music video and 15 min TV video. Materials under the three cultural conditions involve various aspects, such as home decoration, entertainment program music, furniture, costume, cooking, life, film, art and architecture, landscape literature, and so on. Under the American–Chinese fusion culture conditions, American cultural elements and Chinese cultural elements each account for half, and alternate presented (Zhou et al., 2011).

Creative task: Story-writing task. First of all, let the participants read about the story of the Cowherd and the Weaving Maid (a Chinese legend), and then let them to make up a new versions of the story for Turkish children. They are required to try their best to imagine to rewrite the story, make the story more creative, novelty and organization. They not only need to give participants an overview of the original story, but also the information about Turkey's geographical location, climate, religion, economy, industry, and a narrative of everyday Turkish life. Importantly, these participants were unfamiliar with Turkish culture. Before rewriting the story, the subject was asked to report familiarity with Turkish culture, with a score range 1–7. A score of 1 indicating that he was not at all familiar, and a score of 7 indicating that he was very familiar. Finally, the score was *M* = 1.72, *SD* = 0.98.

#### Procedures

First of all, the participants were randomly assigned to four kinds of experimental conditions: (1) the American culture condition: the participants only watched the slide showing about American culture (2) the Chinese culture condition: the participants only watched slides showing about Chinese culture (3) the American–Chinese fusion culture conditions: the participants watched a slide showing of Chinese and American culture alternatively (4) control condition: participants did not watch any power point slides. The slide lasted 45 min, including pictures of music video in the corresponding culture, in which the picture stage was about 20 min, the music stage was about 15 min, and the TV video stage was about 10 min, followed by the subjects finishing the story-writing task.

After the experiment, the four undergraduate students in the department of Chinese language and literature were asked to rate the story making task from the two aspects of novelty and uniqueness (scores ranged from 1 to 7). The novel degree of novelty was about the original story, and the uniqueness was relative to the unique degree among all the stories written. The consistency reliability of novelty was 0.898, the consistency reliability of uniqueness was 0.886.

#### Analysis

SPSS16.0 statistical software was used to test the differences in creative performance under various cultural conditions with one-way ANAVA (Table 1).

**Table 1:** The total score and dimension score of the story-writing task under the conditions of cultural priming.

**Conditions**	**Novelty**			**Originality**			**AUT score**		
	***M***	***SD***	***N***	***M***	***SD***	***N***	***M***	***SD***	***N***
American culture	5.20	0.97	20	4.75	1.16	20	9.95	2.01	20
Fusion culture	4.43	1.38	20	3.93	1.48	20	8.35	2.79	20
Chinese culture	4.45	1.36	20	4.03	1.53	20	8.48	2.78	20
Control group	4.12	1.18	17	3.29	1.34	17	7.41	2.48	17

### Results

The results showed that under all the culture priming conditions, the novelty of the story-writing task reached marginal significance [*F*_(3, 76)_ = 2.656, *p* = 0.055, η_*p*_^2^ = 0.410], and the novelty score of the story-writing task in the United States was significantly higher than that in the control group (*p* = 0.01), and there was no significant difference between the other groups. In addition, there was a significant difference in the originality of the story-writing task under all the culture priming conditions [*F*_(3, 76)_ = 3.437, *p* = 0.021, η_*p*_^2^ = 0.348]. The originality score of American culture groups was significantly higher than that of the control group (*p* = 0.002).

In terms of the total scores of story-writing task under various cultural priming conditions, there were significant differences between the groups [*F*_(3, 76)_ = 3.208, *p* = 0.028, η_*p*_^2^ = 0.333]. The score of the American culture group was significantly higher than that of the control group (*p* = 0.003).

### Discussion

Base on the experimental results, our study suggested that the multicultural experience did have a significant promoting effect on the creativity of the participants, since the performance in American culture priming group was better than the other groups on the whole. However, the promoting effect depended on: (1) whether the participants did get the multicultural experience. The multicultural experience can be formed only when the participants have a certain understanding of the new culture; (2) prototype features contained in multicultural experience. If the multicultural experience included prototypes that promoted creative work, the multicultural experience would promote creativity.

From the study 1, it could be concluded that the significant difference in the creative task was caused by individuals' multicultural experience. When the participants experienced new culture, they might produce culture shock. According to previous studies mentioned above, we could speculate that this culture shock experienced by individuals could improve their attention alertness level and increase the involvement of individuals. Therefore, we would use the biofeedback technology to verify the speculation as previous studies which have shown that individuals' attention alert changes would be reflected in the physiological arousal level.

## Study 2

The participants may have different physiological arousal levels when watching the slides of different cultural conditions, which would have influence on the following creative tasks. This study aimed to explore the relationship between multicultural experience and physiological arousal level.

### Methods

#### Participants

There were three conditions in the experiment: American cultural priming conditions, Chinese cultural priming conditions, American–Chinese fusion culture conditions, and the design of the study was a between-subjects design with 16 subjects in each condition. The subjects had not received biofeedback training in the past, had no surgical or hospitalization experience in the past 1 year, and had no neuropsychiatric history. On the day of the experiment, the subjects could not do strenuous exercise and could not overeat, smoke, drink, or take drugs. The subjects kept an empty stomach for half an hour before the experiment, and the female subjects were not in the Menstrual Cycle. In accordance with the Declaration of Helsinki (1991), the experiment was approved by the Academic Committee of the School of Psychology and the local ethics committee of the School of Psychology, Southwest University in China. We had obtained appropriate ethics committee approval for the research reported, and all subjects gave written informed consent before our experiment.

#### Experimental Materials and Equipments

In order to assess the impact of cultural contents on the participants more objectively, this study adopted the Dutch Spirit 10 biofeedback instrument to collect the physiological indicators of the participants. Physical data, such as skin conductivity (SC), skin temperature (TEMP), electroencephalogram (EEG), blood volume (BVP), electromyography (EMG), and heart rate (HR) were collected. In this experiment, only EEG, SC, TEMP, and HR data were collected. During the experiment, each participant was isolated in a biofeedback laboratory and did not interfere with each other. The subjects could adjust the seat and earphone volume according to the comfort level. During the experiment, the environment was kept quiet.

Creative task: divergent thinking task, in which the subject was asked to write out the alternative uses of a newspaper for 2 min. In study 1, we have proved that experience multicultural could enhance creativity which measurement was story-writing task. As there were so many ways to measure of creativity, so in study 2, we would like to further explore whether we would also conclude the same result when we used other measuring method of the creativity.

Prime materials: priming conditions: American culture, Chinese culture and Chinese-American fusion culture. There are 160 images in the slides and was played with a music background of the corresponding culture for 20 min. After that were 10 min music video and 15 min TV video.

#### Experimental Procedures

First of all, the participants filled in the informed consent form. The experimenters debugged the biofeedback instrument and installed the electrode. Then explained the biofeedback instrument to the participants to reduce the anxiety and discomfort when use of the instrument. Then, baseline levels were measured. The participants sat in a relaxed state for 5 min, and the participants recorded baseline levels of EEG, SC, TEMP, and HR. The mean of the final 3 min measurement was taken as the baseline level. After 5 min, the subjects were shown slides. The physiological indexes of the subjects were recorded when learned the culture. After that, the subjects completed the divergent thinking task within 2 min, and biofeedback data were collected throughout the process.

Spirit 10 biofeedback instrument to collect physiological indexes of EEG, SC, TEMP, and HR. Firstly, participants were required to clean the skin, and then placed electrodes, galvanic skin electrodes were placed in the left hand index finger and ring finger, electrodes placed on the left hand little finger skin temperature. EEG is formulated according to the international society for EEG 10–20 international EEG recording system (Wei and Luo, 2002). Two channels ExG cables were used to conduct the lead connection of the electrodes. Positive 1 was placed at F4, negative 1 at A2, positive 2 at F3, negative 2 at Al, the grounding electrode was placed at Fz, and the blood volume (heart rate) electrode was placed at the middle finger of the left hand.

### Experimental Results and Analysis

#### Physiological Results

Although we recorded the whole physiological indexes during the experiment, we only selected data of a part of each stage in the analysis, and the last 3 min of each stage were taken for analysis (Table 2).

**Table 2:** In the three experimental conditions, participants watched music video and TV video in the baseline stage.

**Indicators**	**Stages**	**American group**			**Fusion group**			**Chinese group**		
		***M***	***SD***	***N***	***M***	***SD***	***N***	***M***	***SD***	***N***
SC	Baseline	0.65	0.06	16	0.62	0.03	16	0.65	0.08	16
	Photograph	0.62	0.05	16	0.61	0.02	16	0.62	0.04	16
	Music	0.66	0.10	16	0.62	0.02	16	0.62	0.04	16
	Video	0.73	0.22	16	0.62	0.02	16	0.63	0.04	16
	After task	0.74	0.21	16	0.63	0.03	16	0.63	0.04	16
TEMP	Baseline	23.05	0.48	16	23.29	0.13	16	23.23	0.18	16
	Photograph	23.20	0.23	16	23.28	0.14	16	23.23	0.18	16
	Music	23.18	0.23	16	23.27	0.15	16	23.22	0.17	16
	Video	23.19	0.24	16	23.26	0.16	16	23.21	0.17	16
	After task	23.04	0.38	16	23.21	0.35	16	23.05	0.34	16
Left θ	Baseline	36.29	12.90	16	25.94	9.41	16	35.79	14.97	16
	Photograph	35.21	13.33	16	32.80	18.40	16	35.62	13.24	16
	Music	30.35	12.50	16	23.94	9.88	16	36.34	30.08	16
	Video	40.36	38.68	16	31.77	16.02	16	37.35	15.94	16
	After task	39.37	16.73	16	38.29	17.92	16	40.05	12.69	16
Left α	Baseline	16.82	5.84	16	13.26	3.13	16	15.21	4.05	16
	Photograph	13.24	3.36	16	14.01	10.83	16	12.45	2.83	16
	Music	11.99	3.12	16	10.16	2.24	16	13.47	10.22	16
	Video	21.21	16.87	16	13.33	8.00	16	14.46	9.48	16
	After task	16.46	6.72	16	17.89	12.66	16	15.90	4.34	16
Left SMR	Baseline	7.74	1.67	16	8.41	3.27	16	7.18	1.91	16
	Photograph	8.29	2.28	16	9.96	11.83	16	7.05	1.51	16
	Music	7.50	1.84	16	6.62	1.69	16	8.24	7.36	16
	Video	11.02	4.83	16	7.94	2.36	16	8.58	2.50	16
	After task	13.52	9.45	16	10.32	4.08	16	9.51	2.55	16
Left β	Baseline	7.34	1.96	16	7.93	3.32	16	6.44	1.41	16
	Photograph	7.93	2.63	16	8.54	8.28	16	6.28	1.65	16
	Music	7.00	1.83	16	6.30	2.04	16	6.84	4.36	16
	Video	10.12	4.36	16	6.96	2.32	16	7.54	2.25	16
	After task	10.61	5.34	16	9.18	3.42	16	8.18	2.14	16
Left γ	Baseline	12.20	5.06	16	13.34	10.70	16	13.77	8.59	16
	Photograph	14.28	7.45	16	15.46	11.47	16	13.59	7.69	16
	Music	16.44	8.14	16	11.39	4.72	16	12.70	5.97	16
	Video	20.12	30.08	16	14.45	7.86	16	14.66	10.22	16
	After task	17.02	9.10	16	14.18	5.87	16	13.25	6.01	16
Right θ	Baseline	36.82	15.14	16	27.28	9.83	16	42.91	35.42	16
	Photograph	37.59	15.74	16	31.68	13.34	16	36.83	15.37	16
	Music	32.99	16.76	16	32.88	23.54	16	31.11	12.09	16
	Video	36.92	12.71	16	30.37	12.45	16	31.84	12.26	16
	After task	39.91	13.99	16	33.44	10.19	16	41.20	12.54	16
Right α	Baseline	12.40	3.52	16	16.10	18.14	16	11.95	3.28	16
	Photograph	12.25	3.23	16	11.73	2.14	16	13.26	3.29	16
	Music	14.59	7.19	16	12.81	3.72	16	13.49	3.81	16
	Video	21.87	3.05	16	13.32	3.22	16	15.58	4.31	16
	After task	16.41	4.79	16	14.04	4.12	16	16.30	4.21	16
Right SMR	Baseline	15.34	2.63	16	7.75	2.96	16	8.72	2.47	16
	Photograph	9.46	6.33	16	7.95	2.95	16	7.75	3.21	16
	Music	7.94	2.67	16	11.26	1.57	16	6.81	1.60	16
	Video	7.54	1.73	16	7.29	1.05	16	7.94	3.12	16
	After task	16.30	4.21	16	8.77	4.09	16	9.62	3.70	16
Right β	Baseline	7.19	2.19	16	11.57	1.88	16	6.02	1.70	16
	Photograph	8.91	6.32	16	7.24	2.62	16	6.78	2.58	16
	Music	10.75	11.05	16	11.67	16.01	16	7.17	2.48	16
	Video	14.74	2.64	16	7.08	2.91	16	7.53	2.25	16
	After task	8.95	2.05	16	7.87	3.61	16	8.13	2.92	16
Right γ	Baseline	17.46	1.69	16	12.60	7.81	16	19.70	2.33	16
	Photograph	18.92	1.92	16	16.56	1.03	16	18.02	1.18	16
	Music	18.32	1.97	16	18.28	1.65	16	15.84	1.53	16
	Video	19.46	1.99	16	14.75	7.19	16	15.61	8.62	16
	After task	14.44	4.09	16	12.60	6.26	16	16.54	15.10	16
HR	Baseline	81.57	24.25	16	82.34	32.53	16	81.95	26.26	16
	Photograph	76.69	25.08	16	66.97	20.51	16	77.03	21.79	16
	Music	85.13	21.85	16	68.75	22.11	16	78.10	17.07	16
	Video	84.03	19.61	16	70.14	25.21	16	73.57	20.19	16
	After task	84.74	27.98	16	83.71	29.43	16	90.45	29.74	16

*In these four stages, the mean and standard deviation of the heart rate of each indicator of the ecg temperature, left alpha, left SMR, right alpha, right alpha, right SMR, right beta, right gamma, were shown in Table 2*.

The results showed that as for the index of SC, the main effects of four stages in each conditions were significant [*F*_(2, 45)_ = 3.557, *p* = 0.037, η_*p*_^2^ = 0.608, *F*_(3, 135)_ = 2.913, *p* = 0.037, η_*p*_^2^ = 0.362] and the interaction effect was also significant [*F*_(6, 135)_ = 2.360, *p* = 0.034, η_*p*_^2^ = 0.403]. In the American group, the SC gradually increased when viewing slide, while in the Chinese group, the SC gradually decreased. Furthermore, there was no significant difference among baseline, photograph and music stages, but in the video stage, the results showed significant difference [*F*_(2, 45)_ = 3.563, *p* = 0.037, η_*p*_^2^ = 0.652]. The American group was significantly higher than the Chinese group (*p* = 0.029) and the fusion group (*p* = 0.022). Under the American culture condition, the four stages of SC had reached marginal significant difference [*F*_(3, 45)_ = 2.556, *P* = 0.067, η_*p*_^2^ = 0.601]. The photograph stage was significantly higher than the video stage (*p* = 0.026). Under the fusion condition, there is no significant difference between the four stages. Under the Chinese culture condition, the four stages also reach marginal significance [*F*_(3, 45)_ = 2.486, *p* = 0.073, η_*p*_^2^ = 0.577]. Furthermore, music stage and baseline stage reached marginal significance (*p* = 0.078).

For each stage of the physiological indexes of three kinds of experimental conditions with one-way ANOVA, the results showed that when considered the left SMR index, At the baseline, photograph and music stages, there was no significant difference between the experimental conditions, but at the video stage, there was significant difference between the three conditions [*F*_(2, 45)_ = 3.594, *p* = 0.036, η_*p*_^2^ = 0.656]. The American group is significantly higher than the Chinese group (*p* = 0.05) and fusion group (*p* = 0.015). Similarly, as for the left β index, there was no significant difference between the experimental conditions in the baseline, photograph and music stages, but at the video stage, there was significant difference between the three conditions [*F*_(2, 45)_ = 4.590. *p* = 0.015, η_*p*_^2^ = 0.775]. The American group is significantly higher than the Chinese group (*p* = 0.025) and fusion group (*p* = 0.007).

#### Results of Creative Tasks and Physiological Indexes at the Task Stage

We analyzed the results of divergent thinking task, and the results are as follows: divergent thinking task is scored from three aspects, including fluency, flexibility and novelty. The index of fluency referred to the number of newspaper uses. The index of flexibility is the category of newspaper use. The score of novelty is to calculate the frequency of occurrence of each item. If it is >10%, 0 points will be recorded; if it is between 5 and 10%, 1 point will be recorded; if it is <5%, 2 points will be calculated (Hu and Adey, 2002). By analyzing the data of the creative test, we found that there is no significant difference in the scores of divergent thinking tasks under the three cultural conditions.

We also recorded biofeedback data as the participants completed their creative tests (Table 2).

The analysis showed that there was significant difference in the SC index in the creative task stage under various cultural conditions [*F*_(2, 45)_ = 4.239, *p* = 0.021, η_*p*_^2^ = 0.608], the American group was significantly higher than the Chinese group (*p* = 0.016) and the fusion group (*p* = 0.015), and there was no difference in other indexes under various cultural conditions.

### Discussion

Electrodermal is the excess or resistance of electrical current between two points in the skin, which measures the activity of the sweat glands and reflects changes in the human physiological state. When individuals' mood is nervous or anxious and wakeful, skin surface sweat, skin conductivity and skin electricity would increase. In a relaxed state, people's mood is calm, sweat gland secretion, skin conductivity, and skin electricity would decrease (Zheng, 2003).

EEG is a kind of rhythmic nerve activity, which could also reflect the physiological arousal state of people. When the brain is highly awakened, the frequency is high. Otherwise, the frequency is low. β waves, frequency range for a 12–36 Hz, appeared when experience mental tension and emotional or stimulated. β waves can be divided into three bands: low Beta waves (12–15 Hz), namely the SMR, middle Beta waves (15–18 Hz), and high Beta waves (12–36 Hz). SMR is related with behaviors, such as analysis judgment, problem solving thinking and listening, middle Beta waves associated with intelligence and mental activity, and high Beta waves is concerned with alert and excited state (Zheng, 2003).

According to the results, the American group was significantly higher in the SC, left SMR and left β than the Chinese group and the fusion group when watching the video. The results showed that the physiological arousal level in American group was relatively high. In addition, the cultural priming conditions in the United States indeed improved the physiological arousal level of the subjects. Finally, arousal level had a significant association with attention. Arousal played an important role in maintaining and changing the excitability of the cerebral cortex and maintaining the state of arousal. It also helped maintaining attention and focusing on consciousness (Xuemin and Xinbao, 2005). This may contribute to the next creative task. In addition, in the video stage, the left-side SMR of the American group was significantly higher than that of the other groups, indicating that the participants showed more analytical problems, thinking, listening and other behaviors when watching video, and their brain activity level was higher. According to Leung and Chiu (2010), researchers believed that multicultural experience promoted creativity because the exposure to multicultural culture expanded individuals' concepts, which enhanced the creative performance of participants. However, the current study considered that conceptual extension was not the only reason why multicultural experience influenced creativity. Confronted with new culture, individuals might be involved in attention switch and the increase of alertness level, which could help individuals to be more engaged in the following creative task and thus improve their creative performance. Previous studies have shown that the improvement of attention alertness level would be accompanied by a series of changes in physiological arousal level. The results of study 2 in this study also confirmed this point and indicated that multicultural experience could increase the individual's attention alertness, which has been reflected in the change of physiological arousal level. Furthermore, previous studies have proved that the increasing of physiological arousal was related to the promotion of creative performance. That was to say, the hypothesis, which suggested that multicultural experience promoted creativity was not only due to the expansion of individual concept, but also the improvement of individual attention alertness level has been indirectly confirmed in study 2. Unfortunately, this study did not produce direct evidence as there was no significant difference in the creative tasks under the three conditions in study 2. We speculated that this was due to a change in creative tasks. In study 1, we used the story-writing task and asked the participants to make up stories for Turkish children. Considering that it was to make up a story for foreign children, in order to make the children understand their stories better, the participants would consider more about cultural differences, so that the promotion of multicultural experience on creative tasks might be more obvious. And the AUT in study 2, although it was a task widely used in the field of creative research, but the task only focus on object uses itself. Essentially, AUT task was a general field of creative task, which was less sensitive to the multicultural experience. Therefore, its promoting effect was weaker, leading to there was no significant difference in creativity performance among three priming conditions. In future studies, we still need to adopt creative tasks which are more related to cultural factors to continue exploring about it. On top of this, it may also be influenced by environmental factors. During the task, the subjects also collected physiological data. As it was a paper-and-pencil test, the electrodes glued on the subjects might have some influence on the writing of the participants, thus affecting the results of the creativity test. In future studies, we should improve on this defect.

## General Discussion

### Reasons Why Multicultural Experiences Influence Creativity

In this study, Chinese college students' creative performance was higher after viewing the slides of American cultural elements than after viewing the slides of Chinese cultural elements and the slides without viewing any cultural elements. It may be that exposure to different cultures gives individuals the ability to perceive more of their inner functions through the surface of things. In addition, this experience may expand the perception scope of participants, activate the upper concept in a semantic network, and expand the concept classification category and concept prototype, so that the individual is easy to break through the mind-set and functional fixation (Yang, 2014). Multicultural experience also exist in problem-solving situations, enabling individuals to extract information automatically from different cultures, and integrate it in novel ways to expanding the conceptual categories in the brain by adopting seemingly unrelated concepts (Ward et al., 1997). For example, when participants were activated to experience multi-racial culture in the context of environmental demands, they could quickly transform among multiple races to improve cognitive flexibility (Gaither S. E. et al., 2015), thus promoting the expansion of creative ideas.

However, the creative task (story-writing task and AUT) adopted in this study was only limited to the verbal divergent thinking task related to semantic concepts, and the performance of the creative task under the American culture priming condition was better than other conditions. This was not completely consistent with the findings of Leung and Chiu. We believed that this result first illustrated the promoting effect of multicultural experience on the creativity of Chinese college students. Chinese college students have grown up under the influence of Chinese culture, and their way of thinking and behavior have been deeply branded with Chinese culture. Exposing to American cultural elements different from Chinese culture would prompt them to compare the differences between the two cultures. Compared with Chinese culture, American culture is more attractive and novel for Chinese college students, because American culture is easy to be associated with open and free innovation. Therefore, the value advocated by American culture is more in line with the characteristics of keeping pace with the times of college students, so. Chinese college students are more interested in American culture and have more contact with it, and their comparison of cultural differences will be more profound. For example, when see a picture of a hamburger, they know that it is fast food in the United States, and also have tasted the taste of it, so, when compare it with the Chinese food, they would not only take it from the color shape and taste, but also can from the ways of production and material. Therefore, they are more likely to get new ideas, extension of the concept of food, such as prototype, they would perform better in divergent thinking task.

However, the American college students in Leung and Chiu's study were not familiar with Chinese culture. Therefore, under the condition of single Chinese culture initiation, they were probably unable to understand Chinese culture with the help of their American cultural knowledge because they actually did not know Chinese cultural elements so that it is impossible to expand the concept and form corresponding concept prototype of something. However, for Chinese college students, why did they perform better in the verbal creative task only under the American culture conditions and there was no difference between fusion condition and Chinese culture condition? We believed that Chinese college students are more familiar with American culture than American students are with Chinese culture, and American culture is more novel and attractive to Chinese students than Chinese culture to American students. Mendelsohn (1976) emphasized the importance of attention process in creative performance and thus influences the following creative tasks. He believed that the difference in individual concentration level is the main reason for the difference in creativity level. Studies by Dewing and Battye (1971); Dykes and Mcghie (1976) and Kasof (1997) showed that individuals with low creativity level had narrower attention than those with high creativity level. So the Chinese students under American culture priming condition would actively analysis the differences between these two cultures due to attention resources were attracted to novel and relatively familiar elements of American culture, so that the concept or concept of something is expanded to form a concept prototype that helps to inspire the next verbal creative task to be completed. However, the Chinese college students under the fusion condition contacted between the two kinds of cultural elements alternatively, so they needed to continue switching between the two kinds of culture. On the one hand, it may extend the participants' concepts of something, and on the other hand, it may also limit the scope of participants' idea or concept extension. Therefore, the performance of verbal creative task is limited. In addition, this study participants were college students under the background of Chinese culture, cultural experience is still the dominant Chinese culture, and the Chinese culture elements under the fusion condition may be a ceiling effect. Besides, the priming effect of American cultural elements is less than half that of American cultural groups in terms of time and quantity. Therefore, the diversity of participants' multicultural experience under American cultural conditions may be superior to that of fusion cultural groups so the performance of the creative task is better than that of the fusion culture group.

### Multicultural Experiences Affect the Physiological Basis of Creativity

In study 2, the participants' galvanic skin gradually rise under the American culture conditions. This may be caused by exposure to new cultural shocks, indicating that the American cultural priming group caused higher physiological arousal of the subjects, and the activation level of the subjects was relatively high, which may have some influence on the following creative tasks. High activation levels can increase the degree of individual attention. In addition, as for the indexes of left SMR and left β in American culture group is higher than other groups. It suggested that the brain activity levels in the left hemisphere were also higher when the subjects viewed a slide of American culture. Some psychologists have shown that the left hemisphere of the brain is involved in continuous processing of analytical language, while the right hemisphere is involved in the overall processing of visual images. Therefore, it is possible that when the subjects watched the American culture slide, they compared and processed the American culture elements and the Chinese culture elements, which expanded the concept of words and words, thus enhancing the activity of the brain.

Finally, there was no difference between the cultural priming groups in the divergent thinking task, which might be caused by the influence of external environmental factors. When the subject was conducting the experiment, the electrode plate was glued on the left hand, which was a certain obstacle to the completion of paper and pencil test, and thus had an impact on the experimental results.

### Deficiencies and Prospects of the Research

In current study we only used photograph, music and video, if we can also provide some information about people how to solve the similar problems in different cultural environment of the material, the multicultural experience obtained at this moment, may not only promote the semantically related creative tasks, such as divergent thinking task, but also help the creative problems like graphical insight problem solving. Of course, it remains to be validated in further research. In addition, the multicultural experience priming conditions used American culture. However, With the increasing economic and cultural exchanges between China and the United States, especially in education of China, American culture is no totally strange for Chinese college students. It remains to be seen whether similar results could emerge if the experimental conditions were changed to a completely unfamiliar culture, or change the participants with less knowledge of American culture. Besides, in the second experiment, creative tasks under the different conditions has no significant difference. It may be because during the paper-pencil tests, the electrodes were glued on the hands of the participants, which had a certain hindering effect on the completion of the task, which should be improved in future experiments.

## Conclusion

The multicultural experiences have a significant promoting effect on the creativity of the subject, but such promoting effect depends on:(1) whether the subject really obtains the multicultural experience; only when the subject has a certain understanding of the new culture can the multicultural experience be formed; (2) the participants acquired the prototype characteristics contained in the multicultural experience. If the multicultural experience included the prototype that had a promoting effect on the creative work, the multicultural experience had a promoting effect on the creativity. In addition, the activation of American culture will increase the physiological arousal level of the individual, increase brain activity, and improve the attention level of the participants, thus contributing to the following creative tasks.

## Author Contributions

LT and GC contributed conception and design of the study. XW performed the statistical analysis and LT wrote the first draft of the manuscript. All authors contributed to manuscript revision, read, and approved the submitted version.

### Conflict of Interest Statement

The authors declare that the research was conducted in the absence of any commercial or financial relationships that could be construed as a potential conflict of interest.

## References

[B1] Amabile T. M. (1996). Creativity in Ontext. Boulder, CO: Westview Press.

[B2] Arjunan S. P. Kumar D. K. Jung T.-P. (2009). Changes in decibel scale wavelength properties of EEG with alertness levels while performing sustained attention tasks. Int. Conf. IEEE Eng. Med. Biol. Soc. 2009, 6288–6291. 10.1109/IEMBS.2009.5332801 19963917

[B3] Aytug Z. G. Rua T. Brazeal D. V. Almaraz J. A. González C. B. (2018). A socio-cultural approach to multicultural experience: why interactions matter for creative thinking but exposures don't. Int. J. Intercult. Relati. 64, 29–42. 10.1016/j.ijintrel.2018.03.004

[B3a] Carlsson I. Wendt P. E. Risberg J. (2000). On the neurobiology of creativity. differences in frontal activity between high and low creative subjects. Neuropsychologia 38, 873–885. 10.1016/S0028-3932(99)00128-1 10689061

[B4] Chang J.-H. Su J. C. Chen H.-C. (2017). Rethinking the multicultural experiences–creativity link: the interactive perspective on environmental variability and dispositional plasticity, in Cambridge Handbook of Creativity and Personality Research, eds Feist G. Reiter-Palmon R. Kaufman J. C. (New York, NY: Cambridge University Press), 124–139. 10.1017/9781316228036.008

[B5] Chang J. H. Hsu C. C. Shih N. H. Chen H. C. (2014). Multicultural families and reative children. J. Cross Cult. Psychol. 45, 1288–1296. 10.1177/0022022114537556

[B6] Chang J. H. Su J. S. Chen H. C. (2015). Cultural distance between parents' and hildren's creativity: a within-country approach in Taiwan. Cult. Divers. Ethnic Minor. Psychol. 21, 477–485. 10.1037/a0037539 25111548

[B7] Chua R. Y. J. Morris M. W. Mor S. (2012). Collaborating across cultures: cultural metacognition and affect-based trust in creative collaboration. Organ. Behav. Hum. Decis. Process. 118, 116–131. 10.1016/j.obhdp.2012.03.009

[B8] Chua R. Y. J. Roth Y. Lemoine J.-F. (2014). The impact of culture on creativity how cultural tightness and cultural distance affect global innovation crowdsourcing work. Adm. Sci. Q. 60:0001839214563595 10.1177/0001839214563595

[B9] Clark L. A. Mcewen J. L. Collard L. M. Hickok L. G. (1993). Symptoms and traits of personality disorder: two new methods for their assessment. Psychol. Assess. 5, 81–91. 10.1037/1040-3590.5.1.81

[B10] Csikszentmihalyi M. (1996). Creativity: Flow and the Psychology of Discovery and Invention. New York, NY: HarperCollins.

[B11] De Dreu C. K. W. (2010). Human creativity: reflections on the role of culture. Manage. Organ. Rev. 6, 437–446. decision processes, 118: 116–131. 10.1111/j.1740-8784.2010.00195.x

[B12] Dewing K. Battye G. (1971). Attention deployment and non-verbal fluency. J. Pers. Sicial Psychol. 17, 214–218.

[B13] Dong Y. Bartol K. M. Zhang Z.-X. Li C. (2016). Enhancing employee creativity via individual skill development and team knowledge sharing: influences of dual-focused ransformational leadership. J. Organ. Behav. 38, 439–458. 10.1002/job.2134

[B14] Dykes M. Mcghie A. (1976). A comparitive study of attentional strategies of schizophrenic and highly creative normal subjects. Br. J. Psychiatry 128, 50–56. 1252702 10.1192/bjp.128.1.50

[B15] Falavarjani M. F. Irandust F. (2017). Does exposure to multicultural experience enhance all individuals' creative problem-solving ability? Int. J. Soc. Sci. Res. 5:14 10.5296/ijssr.v5i2.11173

[B16] Fink A. Koschutnig K. Hutterer L. Steiner E. Benedek M. Weber B.. (2014). Gray matter density in relation to different facets of verbal creativity. Brain Struct. Funct. 219, 1263–1269. 10.1007/s00429-013-0564-0 23636224

[B17] Gaither C. J. Roberts N. S. Hanula K. L. (2015). Visitor Diversity Through the Recreation Manager Lens: Comparing Forest Service Regions 8 (us South) and 5 (California). General Technical Report - Southern Research Station, USDA Forest Service.

[B17a] Gaither S. E. Cohen-Goldberg A. M. Gidney C. L. Maddox K. B. Gidney C. L. Gidney C. L. (2015). Sounding black or white: priming identity and biracial speech. Front. Psychol. 6:457. 10.3389/fpsyg.2015.00457 25941505 PMC4403599

[B18] Galinsky A. D. Maddux W. W. Ku G. (2006). The view from the other side of the table: getting inside your counterpart's head can increase the value of the deal you walk away with. Here's how to do it. Negotiation 9, 1–5.

[B19] Guimerà R. Uzzi B. Spiro J. Amaral L. A. N. (2005). Team assembly mechanisms determine collaboration network structure and team performance. Science 308, 697–702. 10.1126/science.1106340 15860629 PMC2128751

[B20] Hu S. Gu J. Liu H. Huang Q. (2017). The moderating role of social media usage in the relationship among multicultural experiences, cultural intelligence, and individual creativity. Inform. Technol. People 30, 265–281. 10.1108/itp-04-2016-0099

[B21] Hu W. Adey P. A. (2002). Scientific creativity test for secondary school student. Int. J. Sci. Educ. 24, 384–403. 10.1080/09500690110098912

[B22] Hudspith M. John G. R. Nhamburo P. T. Littleton J. M. (1985). Effect of ethanol *in vitro* and *in vivo* on Ca^2+^-activated metabolism of membrane phospholipids in rat synaptosomal and brain slice preparations. Alcohol 2, 133–138. 10.1016/0741-8329(85)90030-8 2861830

[B23] Jausovec N. Jausovec K. (2000). Eeg activity during the performance of complex mental problems. Int. J. Psychophysiol. 36, 73–88. 10.1016/S0167-8760(99)00113-0 10700625

[B24] Junaid S. Pertiwi I. (2017). Culture shock experienced by main character in Lauren Kate's novel “Torment” by using the psychology of culture shock by Collen Ward. English Literature J. 2, 108–125.

[B25] Kasof J. (1997). Creativity and breadth of attention. Creat. Res. J. 10, 303–315.

[B26] Kristian G. (2013). The Bleeding Land. Hijos Del Trueno.

[B27] Kurpis L. H. Hunter J. (2016). Developing students' cultural intelligence through an experiential learning activity a cross-cultural consumer behavior interview. J. Market. Educ. 39:0273475316653337 10.1177/0273475316653337

[B28] Lambert W. E. Tucker G. R. d'Anglejan A. (1973). Cognitive and attitudinal consequences of bilingual schooling: the St. Lambert project through grade five. J. Educ. Psychol. 65, 141–159.

[B29] Leung A. K. (2008). Multicultural experience enhances creativity: the when and how. Am. Psychol. 63, 169–181. 10.1037/0003-066X.63.3.169 18377107

[B30] Leung A. K. Y. Chiu C. Y. (2010). Multicultural experience, idea receptiveness, and creativity. J. Cross Cult. Psychol. 41, 723–741. 10.1177/0022022110361707

[B31] Martindale C. Anderson K. Moore K. West A. N. (1996). Creativity, oversensitivity, and rate of habituation. Pers. Indiv. Differ. 20, 423–427. 10.1016/0191-8869(95)00193-X

[B32] Martindale C. Hasenfus N. (1978). EEG didderences as a function of creativity, stage of the creative process, and effort to be oringinal. Biol. Psychol. 6, 157–167. 667239 10.1016/0301-0511(78)90018-2

[B33] Mendelsohn G. A. (1976). Associative and attentional processes in creative performance. J. Pers. 44, 341–369.

[B34] Miron-Spektor E. Beenen G. (2015). Motivating creativity: the effects of sequential and simultaneous learning and performance achievement goals on product novelty and usefulness. Organ. Behav. Hum. Decis. Process. 127, 53–65. 10.1016/j.obhdp.2015.01.001

[B35] Oken B. S. Salinsky M. C. Elsas S. M. (2006). Vigilance, alertness, or sustained attention: physiological basis and measurement. Clin. Neurophysiol. 117, 1885–1901. 10.1016/j.clinph.2006.01.017 16581292 PMC2865224

[B36] Pfaff D. W. (2008). Molecular and biophysical mechanisms of arousal, alertness, and attention. Ann. N. Y. Acad. Sci. 1129:xi. 10.1196/annals.1417.034 18591463

[B37] Presbitero A. (2016). Culture shock and reverse culture shock: the moderating role of cultural intelligence in international students' adaptation. Int. J. Intercult. Relat. 53, 28–38. 10.1016/j.ijintrel.2016.05.004

[B38] Razumnikova O. M. (2007). Creativity related cortex activity in the remote associates task. Brain Res. Bull. 73, 96–102. 10.1016/j.brainresbull.2007.02.008 17499642

[B39] Runco M. A. Jaeger G. J. (2012). The standard definition of creativity. Creat. Res. J. 24, 92–96. 10.1080/10400419.2012.650092

[B40] Sawyer K. (2006). Explaining Creativity: The Science of Human Motivation. New York, NY: Oxford University Press.

[B41] Shi D. Liu L. Ke Y. (2012). The influence of multicultural experience on creative problem solving. China Sci. Educ. Innov. Guide 29, 16–18. 10.3969/j.issn.1673-9795.2012.29.014

[B42] Simonton D. K. (1999). Origins of Genius: Darwinian Perspectives on Creativity. New York, NY: Oxford University Press.

[B43] Smolders K. C. H. J. de Kort Y. A. W. (2014). Bright light and mental fatigue: effects on alertness, vitality, performance and physiological arousal. J. Environ. Psychol. 39, 77–91. 10.1016/j.jenvp.2013.12.010

[B44] Sparkman D. J. Eidelman S. Blanchar J. C. (2016). Multicultural experiences reduce prejudice through personality shifts in openness to experience. Eur. J. Soc. Psychol. 46, 840–853. 10.1002/ejsp.2189

[B45] Sternberg R. J. Amabile T. M. Lubart T. I. (1999). Handbook of Creativity. New York, NY: Cambridge University Press.

[B46] Tendayi Viki G. Williams M. L. J. (2014). The role of identity integration in enhancing creativity among mixed-race individuals. J. Creat. Behav. 48, 198–208. 10.1002/jocb.48

[B47] Ward T. B. Smith S. M. Vaid J. (1997). Conceptual structures and processes in creative thought, in Creative Thought: An Investigation of Conceptual Structures and Processes, eds T. B. Ward, S. M. Smith, and J. Vaid (Washington, DC: American Psychological Association), 1–27.

[B48] Wei J. Luo Y. (2002). A Course of Cognitive Event-Related Brain Potentials. Beijing: Economic Daily Press.

[B49] Wyspianski J. O. Barry W. F. Dayhaw L. T. (1963). Brain Wave Amplitude and Creative Thinking. Revue de l'Universite d' Ottawa, 260–276.

[B50] Xuemin Z. Xinbao W. (2005). Influence of arousal level on tennis. J. Jiujiang Univ. 20, 120–122.

[B51] Yang X. Li H. (2015). Research review on the value of multicultural experience. Contemp. Educ. Cult. 4, 26–32. 10.3969/j.issn.1674-5779.2015.04.005

[B52] Yang Y. (2014). The Influence of Multicultural Experience on the Way of Information Processing. Doctoral dissertation.

[B53] Yang Y. Wan M. (2012). New progress in creativity research: the influence of multicultural experience on creativity. Contemp. Educ. Cult. 4, 86–91. 10.3969/j.issn.1674-5779.2012.05.016

[B54] Zha P. Walczyk J. J. Griffith-Ross D. A. Tobacyk J. J. (2006). The impact of culture and individualism-collectivism on the creative potential and achievement of American and Chinese adults. Creat. Res. J. 18, 355–366. 10.1207/s15326934crj1803_10

[B55] Zheng Y. (2003). Clinical Practice of Biofeedback. Beijing: Advanced Education Press.

[B56] Zhou H. Zhang Q. (2002). New progress in creative physiological research. Exploration of Psychol. 22, 9–13. 10.3969/j.issn.1003-5184.2002.03.002

[B57] Zhou T. Cao G. Zhou S. (2011). Different Culture Priming Lead to Different Creative Performance. Chongqing: Scientific Research.

